# Long-Term Stability of Nicotinamide Cofactors in Common Aqueous Buffers: Implications for Cell-Free Biocatalysis

**DOI:** 10.3390/molecules29225453

**Published:** 2024-11-19

**Authors:** Kody D. Wolfe, Markus Alahuhta, Michael E. Himmel, Yannick J. Bomble, G. Kane Jennings, David E. Cliffel

**Affiliations:** 1 Institute for Sustainable Energy & The Environment, Ohio University, Athens, OH 45701, USA; wolfek1@ohio.edu; 2 National Renewable Energy Laboratory, Biosciences Center, Golden, CO 80401, USA; petri.alahuhta@nrel.gov (M.A.); mike.himmel@nrel.gov (M.E.H.); yannick.bomble@nrel.gov (Y.J.B.); 3 Chemical & Biomolecular Engineering Department, Vanderbilt University, Nashville, TN 37235, USA; 4 Chemistry Department, Vanderbilt University, Nashville, TN 37235, USA

**Keywords:** NADH, NAD^+^, Tris, phosphate, HEPES, enzymatic, catalysis, degradation

## Abstract

The use of nicotinamide cofactors in cell-free biocatalytic systems is necessitated by the high specificity that these enzymes show for their natural redox mediators. Unfortunately, isolation and use of natural cofactors is costly, which suggests that enhancing their stability is key to enabling their use in industrial processes. This study details NAD^+^ and NADH stability in three buffer systems (sodium phosphate, HEPES, and Tris) at 19 °C and 25 °C and for up to 43 d. In Tris, both NADH and NAD^+^ were found to be highly stable. NADH degradation rates of 4 μM/d (19 °C) and 11 μM/d (25 °C) were observed in Tris buffer, corresponding to >90% and 75% remaining after 43 d, respectively. Higher degradation rates (up to 34 μM/d) were observed when sodium phosphate or HEPES buffers were used. The effect of a mild increase in temperature was determined to be significant for long-term stability, and it was shown that degradation under these conditions can be easily monitored via UV–Vis, because the degradation proceeds via the oxidation/de-aromatization of the dihydropyridine ring. Overall, this work emphasizes that the choice of buffer system is consequential for bioreactor systems employing natural nicotinamide cofactors for extended periods of time.

## 1. Introduction

Enzymatic cofactor regeneration is considered a potential solution to feeding costly cofactors to cell-free bioreactors [1,2,3,4]. A recent review by Sharma et al. quantified potential regeneration schemes and outlined barriers to successful implementation [5]. One such barrier is the stability of nicotinamide adenosine dinucleotide cofactors (NAD^+^ and NADH) in solution when considering the possibility of long-term cofactor regeneration in cell-free systems [6,7,8,9,10,11,12,13]. Unfortunately, NAD(H) cofactors have complex degradation considerations [6,7,14,15]. Specifically, the oxidized form (NAD^+^) undergoes base-catalyzed degradation at high pH, whereas the reduced form (NADH) undergoes acid-catalyzed degradation at low pH. Chenault and Whitesides investigated the stability of nicotinamide cofactors [14], showing that the NAD^+^/NADH system has an optimum pH of approximately 8.5 with respect to minimizing the degradation of both species. The total first-order rate constant of NADH degradation ($k_{obs}$) is given as

(1) $$k_{obs}=k_{w}+k_{H+}+k_{HA}$$

where $k_{w}$ is the rate constant for general degradation, $k_{H+}$ is the rate constant for general acid-catalyzed degradation, and $k_{HA}$ is the rate constant for specific acid-catalyzed degradation [14]. A similar equation may be written for the base-catalyzed degradation of NAD^+^.

General degradation ($k_{w}$) is predominantly a function of temperature, whereas acid-catalyzed degradation ($k_{H+}$) is dependent on the pH of the solution. Specific acid-catalyzed degradation ($k_{HA}$) is a function of the solution pH, the specific acid in use, and the pKa and concentration of the specific acid (HA), which is typically the conjugate acid of the buffer [7,12,13]. Therefore, buffers with high pKa values result in a lower concentration of HA and reduce the rate of NADH degradation. Conversely, the degradation of NAD^+^ is base-catalyzed, and the opposite trend holds true. The generally opposite trend of NAD^+^ and NADH stability as a function of pH necessitates finding a balance in their respective degradation rates in systems where the stability of both species is necessary, such as the case in cell-free bioreactors that employ a cofactor regeneration scheme [1,2,3,14,16,17,18].

**Figure 1:**
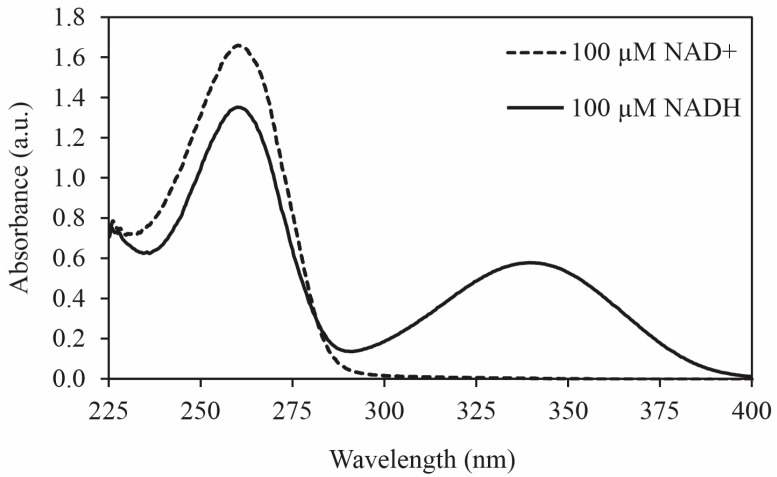
Absorbance spectra of 100 μM NAD^+^ and 100 μM NADH in HEPES buffer pH 8.5. The absorbance peak at 340 nm is attributed to the reduced dihydropyridine ring of NADH.

The optimal conditions for solutions containing NAD^+^ and NADH have been separately reported as ~pH 8.5, room temperature or cooler, and biologically relevant buffers with high pKa values, such as HEPES and Tris buffers, whereas phosphate-containing buffers have been shown to have high rates of specific acid-catalyzed degradation of NADH [6,7,8,9,12,13,15,18]. Methods used in previous reports for studying NAD^+^/NADH degradation were largely based upon UV–Visible spectroscopy and spanned a timeframe relevant to the study underway, which is much shorter than that necessary for a continuous bioreactor application [9,10,12]. The reduced form (NADH) contains a dihydropyridine ring that absorbs strongly at both 260 and 340 nm, whereas the oxidized form (NAD^+^) contains a pyridine ring that absorbs at 260 nm and does not absorb at 340 nm (Figure 1) [9,12].

**Figure 2:**
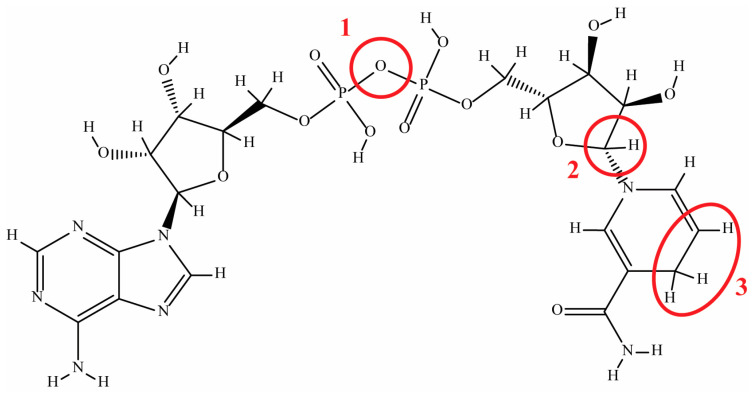
Structure of NADH with the likely locations of degradation labeled: (1) phosphate–phosphate linkage, (2) nicotinamide–ribose linkage, and (3) carbons C_5_ and C_6_ of the dihydropyridine ring (subject to acid-catalyzed hydrolysis and nucleophilic attack).

Figure 2 shows the three likely locations of degradation of NADH: (1) phosphate–phosphate linkage, (2) nicotinamide–ribose linkage, and (3) carbons C_5_ and C_6_ of the dihydropyridine ring [6,19]. Various degradation mechanisms have been reported with multiple reactions occurring sequentially and on varying time scales [19]. The likely degradation products include D-ribose 5-phosphate, nicotinamide, adenylic acid or adenosine monophosphate, and nicotinamide mononucleotide [13,19]. Effort has been made to improve the stability of nicotinamide cofactors in aqueous solutions, including the use of organic, inorganic, and biological additives, as well as modification of the pyridine ring and development of engineered cofactor alternatives or biomimetics [10,17,20,21,22,23,24,25,26]. The work presented here is aimed at determining the optimum buffer conditions for long-term stability of both NAD^+^ and NADH, upon which other, more complex methods may be developed to further improve the stability.

Previous studies of nicotinamide cofactor stability have focused on buffer effects for specific applications or studied degradation at high-temperature expedite degradation [8,10,12,13,18]. Based on these reports, conditions were chosen for the study reported here that should result in long-term stability, necessary for the development of an effective NADH regeneration system. Surprisingly, numerous recent studies of NADH regeneration schemes utilized buffer systems that have been reported to rapidly degrade the cofactor [27,28,29,30,31,32]. The choice of buffer system in those studies was likely multifaceted and may have included considerations for the specific enzymes used, buffer availability at a large scale, or buffers traditionally used for specific fields of biocatalysis. To provide a comprehensive scope of NAD^+^/NADH stability, three commonly reported buffers were studied in this work.

To study the long-term stability necessary for cell-free biocatalysis, a 43-d degradation study was conducted. The buffers used were 50 mM 2-amino-2-(hydroxymethyl)propane-1,3-diol (Tris), 4-(2-hydroxyethyl)-1-piperazineethanesulfonic acid (HEPES), and sodium phosphate at pH 8.5. In addition to the typical UV–Visible spectroscopic measure of stability, an enzymatic assay to show proof of biological activity was also performed. The results show that Tris buffer is an attractive buffer system for the NAD^+^/NADH system, because it exhibits long-term cofactor stability. These findings show that the choice of buffer system is critical for the multiday operation of cell-free biocatalytic reactors that employ NAD^+^/NADH cofactor regeneration.

## 2. Results

### 2.1. NADH Stability and UV–Visible Spectra

**Figure 3:**
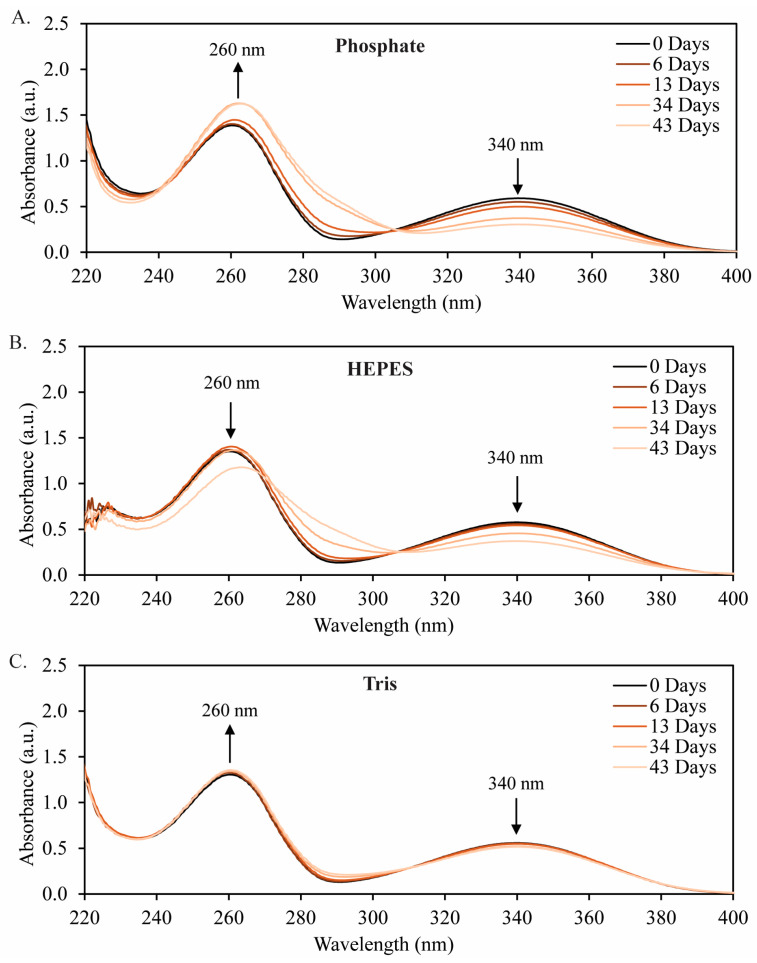
UV–Vis spectra for 2 mM NADH in (**A**) sodium phosphate buffer, (**B**) HEPES buffer, and (**C**) Tris buffer over a span of 43 d. The peak at 340 nm is assigned to dihydropyridine (characteristic of NADH), and the peak at 260 nm is assigned to pyridine (characteristic of NAD^+^).

**Figure 4:**
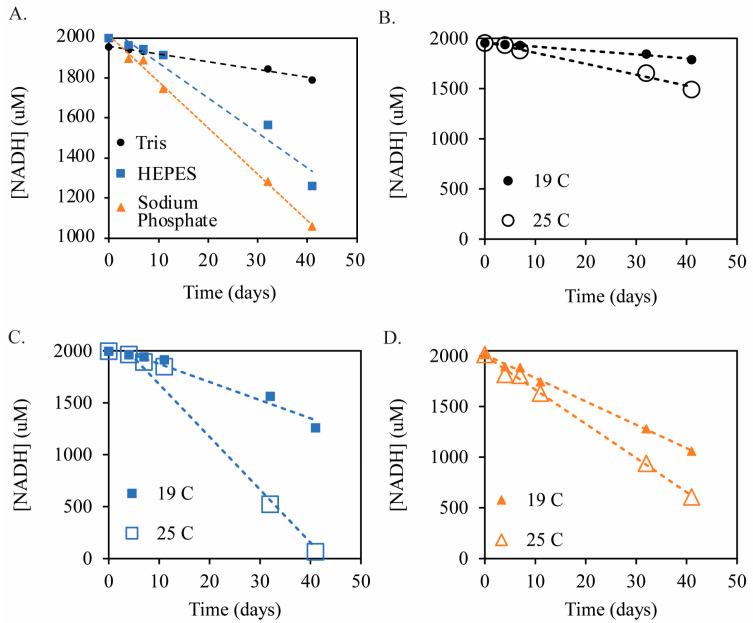
Stability of NADH in Tris buffer, HEPES buffer, and phosphate buffer is shown in (**A**). For each buffer, the pH was ~pH 8.5 and the temperature was 19 °C. The initial concentration of NADH was 2 mM, and the stability was measured using the absorbance of NADH at 340 nm and referencing the calibration curve made for each buffer. The effect of a 6 °C increase in temperature on the degradation rate of NADH is shown for each buffer: (**B**) Tris, (**C**) HEPES, and (**D**) sodium phosphate buffers. Note that the 11-day data point in Tris buffer was determined to be erroneous and was omitted.

For the initial UV–Visible spectroscopic stability study, 340 nm absorbance was used to track the concentration of NADH in each buffer over time (Figure 3). The measured concentration of NADH did not change appreciably in any of the three buffers over the first six days at pH 8.5 and 19 °C. However, over several weeks of testing under these conditions, Figure 3 shows that NADH was most stable in Tris buffer but degraded more rapidly in HEPES and most rapidly in phosphate buffer. The absorbances in Figure 3 are plotted with time for the three buffers in Figure 4A to determine the rate of NADH degradation in each buffer at pH 8.5 and 19 °C. The rate of degradation increases in the following order: Tris (4 μM/day) < HEPES (18 μM/day) < phosphate (23 μM/day). Therefore, the rates of degradation of NADH in HEPES and phosphate buffers are higher by 4.5 and 5.8 times, respectively, than that measured when using the Tris buffer.

### 2.2. Effect of Temperature on NADH Degradation

The effect of temperature on the degradation of NADH was also investigated. Samples of 2 mM NADH in 50 mM buffer at pH 8.5 were stored in a water bath at 25 °C. The elevated temperature (+6 °C) increased the rate of degradation in each of the three buffers tested (Figure 4B–D). The degradation rates increased from 4 μM/d to 11 μM/d in Tris buffer (Figure 4B), 18 μM/d to 51 μM/d in HEPES buffer (Figure 4C), and 23 μM/d to 34 μM/d in sodium phosphate buffer (Figure 4D). These results show that even a mild increase in temperature has a significant effect on NADH stability. At 19 °C, over 90% of the NADH is retained over 40 d in Tris buffer, whereas, at 25 °C, only 75% of the NADH remains. To summarize the key points in Figure 3 and Figure 4, 50 mM Tris buffer at pH 8.5 and lower temperatures (19 °C) are ideal conditions for the long-term stability of NADH in solution.

### 2.3. Qualitative Study of NAD^+^ Degradation

**Figure 5:**
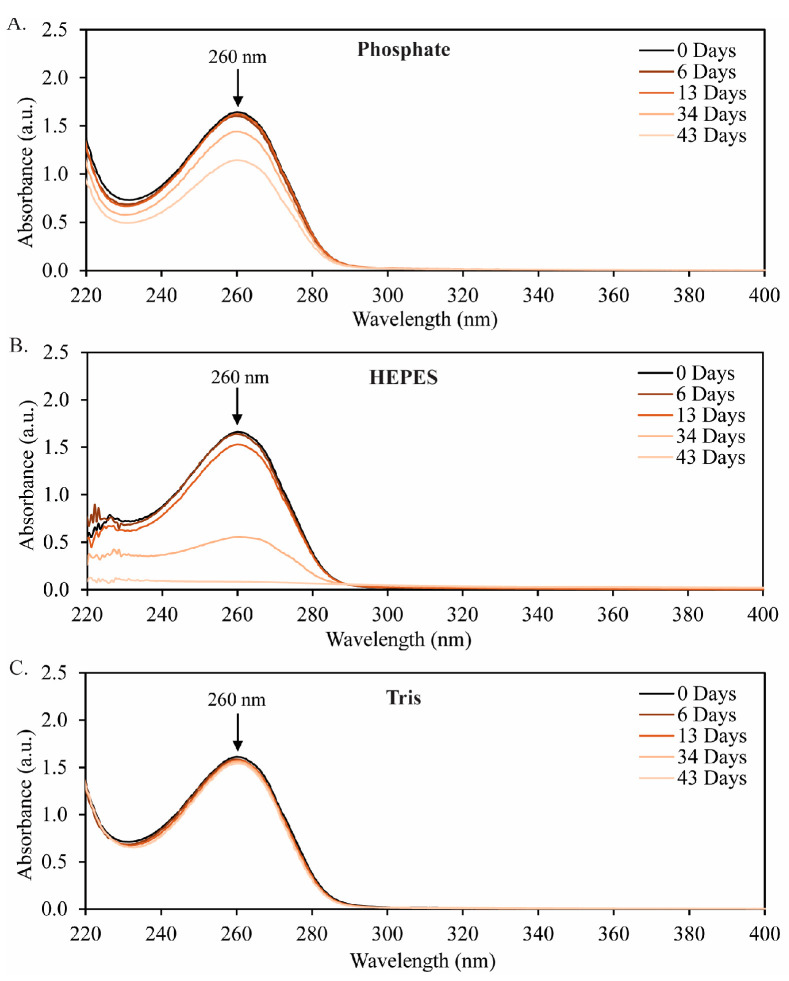
Stability of NAD^+^ in (**A**) phosphate buffer, (**B**) HEPES buffer, and (**C**) Tris buffer over 43 days. The initial concentration was 2 mM NAD+, and the buffers were prepared at pH 8.5 and stored at 19 °C. The decrease in peak intensity at 260 nm is attributed to the degradation of NAD^+^, but the degradation rate cannot be precisely determined from these data due to the inability to generate an accurate calibration curve for NAD^+^-only solutions.

**Figure 6:**
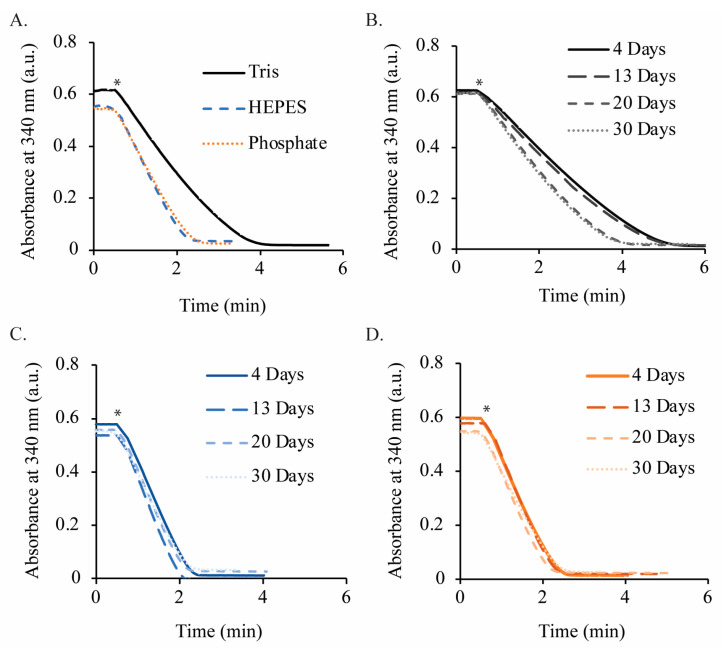
Enzymatic activity testing data over 30 d for 100 μM NADH solutions in each buffer. (**A**) The 30-day curve for each buffer. Assay curves taken at intervals throughout the study are shown for (**B**) Tris, (**C**) HEPES, and (**D**) phosphate buffers. All buffer concentrations are 50 mM, pH 8.5, and solutions are stored at 19 °C. Enzymatic activity was tested by spiking a dilute solution of NADH oxidase (NOX) into the cuvette at t = 0.5 min (marked by an asterisk) and measuring the decrease in absorbance at a 340 nm wavelength.

The direct determination of the concentration of NAD^+^ in solution is not straightforward due to the potential of NADH degradation products (mainly acid-catalyzed products with a hydroxyl addition on the dihydropyridine ring) also absorbing at 260 nm [9,12]. As such, our analysis of stability for NAD^+^ is meant to be qualitative. Therefore, to qualitatively study the degradation of NAD^+^, the absorbance spectra for 2 mM NAD^+^ was measured in each of the three tested buffer systems over 43 d (Figure 5). First, we will discuss the behavior in sodium phosphate buffer (Figure 5A). The reduction in the peak absorbance at 260 nm indicates that NAD^+^ degrades at a similar rate to NADH over the 43-day experiment in phosphate buffer. In HEPES buffer (Figure 5B), the 2 mM NAD^+^ solution degraded nearly entirely over the 43-d period. Thus, while NADH is more stable in HEPES buffer than in sodium phosphate, the oxidized cofactor (NAD^+^) degrades more rapidly in HEPES. There is also a clear red shift in the 260 nm peak as the NAD^+^ degrades. Lastly, in Tris buffer (Figure 6C), the NAD^+^ peak at 260 nm slowly and slightly decreases (by 4%) over the course of the experiment. Overall, the spectra show that the degradation of NAD^+^ in Tris buffer is much slower than the degradation in phosphate or HEPES buffers.

### 2.4. Validation of Enzymatic Activity

The often-used UV–Visible spectroscopic method for NAD^+^/NADH quantification can provide false-positive stability information, because degradation products may contain either dihydropyridine or pyridine, thereby resulting in absorbance at 340 or 260 nm, respectively [9,12,13]. To test for biologically inactive degradation products, the stability results discussed above were followed by an enzymatic activity stability study that employed a NADH oxidase (NOX). First, Figure S2 shows full spectrum examples of the consumption of freshly prepared 100 μM NADH (150 μL of 2 mM NADH stock solution diluted to 3 mL in the cuvette by NOX) in each buffer. Interestingly, Tris buffer yields the slowest NOX reaction rate, whereas NOX in HEPES and phosphate buffers exhibits a similarly higher reaction rate. The slower reaction rate in Tris buffer may be indicative of interactions between the Tris buffer with either NOX or NADH. Interactions between Tris buffers and enzymes have been previously reported but are specific to particular enzymatic systems and thus may necessitate a tailored study for a cell-free biocatalytic scheme that wishes to employ Tris buffer [33,34,35].

The metric for the enzymatic activity test is the ratio of the absorbance at 340 nm after the enzymatic reaction has completed in comparison to the absorbance at the beginning of the test. If a significant absorbance is still observed after the reaction, then non-enzymatically active NADH (containing a dihydropyridine moiety) is present in the sample. The enzymatic activity-based stability study of NADH over 30 d, as determined via the NOX assay, is shown for the three buffers in Figure 6. An aliquot of each solution was removed, and the NOX assay was performed at 4, 13, 20, and 30 d. In each buffer, the absorbance was reduced to a near-zero value after several minutes, indicating that any remaining NADH was consumed by the NOX enzyme and is therefore considered to be biologically active. The lower stability of NADH can be seen at the y-intercept for the phosphate (Figure 6B) and HEPES (Figure 6C) buffers, whereas the y-intercept value remains stable for Tris buffer (Figure 6D). This data set is complementary to the previous stability analyses and demonstrates that the improved stability in Tris buffer at pH 8.5 (in comparison to HEPES or phosphate buffers) also preserves the enzymatic activity. If inactive NADH degradation products containing an intact dihydropyridine ring were present, the absorbance at 340 nm would not have dropped to zero or near-zero values. Therefore, because NOX was able to consume the remaining NADH over 30 d in each buffer, we conclude that the final degradation products do not contain dihydropyridine and that any remaining absorbance at 340 nm corresponds to active NADH. Thus, the NADH degradation rates shown in Figure 3 and Figure 4 can be interpreted as enzymatically active NADH degradation rates over a long-term, multi-week study.

## 3. Discussion

The NADH stability studies conducted at 19 and 25 °C (Figure 3 and Figure 4) show that, although NADH is stable for several days in each buffer, if long-term use or storage is required, Tris buffer yields the slowest degradation rate (4 μM/day). The degradation rates of NADH in HEPES and sodium phosphate buffer are four to six times greater than in Tris buffer at 19 °C. Further, a mild increase in temperature (25 °C) more than doubled the degradation rates in the Tris and HEPES buffers and nearly doubled the rate in sodium phosphate. The activation energy, $E_{a}\;\left(\frac{J}{mol}\right)$, is directly related to the reaction rate constant, $k\;\left(\frac{mol}{s}\right)$, temperature, T (K), and the ideal gas constant, $R=8.314\frac{J}{mol.K}$, via the Arrhenius equation, where A is a pre-exponential factor:

(2) $$k=A\times {exp}^{\left(\frac{-E_{a}}{RT}\right)}$$

An analysis of the activation energies of degradation in each buffer was performed using a linearized Arrhenius equation fit to the rates of degradation at each temperature, with ln(k) as the dependent variable, $\frac{-1}{RT}$ as the independent variable, ln(A) as the y-intercept, and $E_{a}$ as the slope:

(3) $$\ln\left(k\right)=\frac{-E_{a}}{RT}+ln(A)$$

A significantly lower activation energy was found for phosphate buffer (46 kJ/mol) in comparison to Tris and HEPES (125 and 128 kJ/mol, respectively). The distinctly lower activation energy in phosphate buffer suggests a catalyzed degradation mechanism, possibly via the formation of a phosphate–pyridine adduct, as reported previously [12,13]. The strong effect of temperature on the degradation rate delineates this work from previously reported stability tests at extreme temperatures with the goal of exhibiting highly stable NADH solutions [13].

The stability of NAD^+^ was qualitatively monitored throughout the 43-day stability tests, and Tris buffer was again found to be the most stable buffer system. Also, in the case of NAD^+^, HEPES buffer was found to completely degrade the cofactor over the 43-day experiment. Further studies, using quantitative methods such as LC-MS, would be necessary to determine the root cause of the high degradation of NAD^+^ in HEPES. Additionally, more complex degradation studies, including both cofactors in solution, may be warranted for a better understanding of how systems employing cofactor generation should be designed, because both the oxidized and reduced forms will require long-term stability [4].

The NOX enzymatic activity assay showed that, after 30 days, all remaining NADH that was detectable via UV–Vis was indeed biologically active. This result is critical, because it shows that degradation of NADH in the reported buffers results in degradation products with either an oxidized or degraded dihydropyridine ring (Figure 2). The degradation mechanism in the chosen buffers thus lends itself to facile monitoring of NADH in a cell-free biocatalytic system, because UV–Vis may be used for real-time monitoring of the NADH concentration. The final degradation products under various conditions have been reported and agree with the general conclusions stated here [6,13,19]. In summary, long-term NADH/NAD^+^ stability can be achieved near room temperature and may be applied to either cofactor storage or the operation of cell-free biocatalytic systems.

## 4. Materials and Methods

### 4.1. UV–Visible Spectroscopy for NADH Determination

The dihydropyridine ring of NADH can be observed via absorbance measurement at a wavelength of 340 nm (NAD^+^ does not absorb at 340 nm). Stability test solutions were diluted in the cuvette with the buffer specific to the solution being tested. The absorbance maximum at 340 nm was checked against NADH calibration curves (Figure S1). The 100 μM NADH (cuvette concentration) absorbs approximately 0.58 a.u. at 340 nm. Reference cuvette solutions were equally diluted aliquots of the relevant buffer (without NAD^+^/NADH) in deionized water.

### 4.2. UV–Visible Stability Study

Sample solutions were prepared with a range of NADH concentrations from 0 to 2000 μM, with the total NAD^+^/NADH concentration held constant at 2000 μM (i.e., 800 μM NADH and 1200 μM NAD^+^). The buffer conditions were pH 8.5, 19 °C, and a total buffer concentration of 50 mM. UV–Vis calibration curves were taken at t = 1 h from wavelengths of 400 nm to 200 nm using 0 to 100 μM NADH solutions (Figure S1). Note that the slope varied slightly between the buffers. The calibration curves determined for each buffer were used to determine the concentration of NADH in the respective buffers during stability testing. Absorbance spectra were collected at a 19:1 dilution of the stock solutions and at various time intervals spanning approximately two weeks to determine the time-dependent concentration of NADH.

### 4.3. Enzyme Expression and Purification

*Lactobacillus brevis* NADH oxidase (NOX) was used to verify the biological activity of NADH after prolonged incubation with the buffers (amino acid sequence is given in the Supporting Information). NOX was cloned into the pET28 vector with a C-terminal his-tag and expressed in *E. coli* in 1.5 L of lysogeny broth (LB) media using a shaking incubator controlled at 225 rpm and 37 °C until OD_600_ 0.6 and 0.8 was reached. The culture was then cooled to 18 °C on ice for 15 min before induction with 0.3 mM IPTG. After 18 h of induction, cell pellets were collected by centrifugation at 8000× *g* for 15 min and stored at −20 °C. The cell pellets were lysed by thawing in equal volume of His A buffer (50 mM Tris buffer pH 7.5, 500 mM NaCl, and 10 mM imidazole) with >5 mg/mL of lysozyme from hen egg white (Sigma-Aldrich Corp., St. Louis, MO, USA), Pierce^TM^ Protease Inhibitor (Thermo Fisher Scientific, Waltham, MA, USA), and 500 U Pierce^TM^ Universal Nuclease (Thermo Fisher Scientific, Waltham, MA, USA). This mixture was then rotated at room temperature for 30 min. The lysate was then freeze-thawed twice with liquid nitrogen and a 35 °C water bath to complete the cell lysis and then cleared by centrifugation at 14,000× *g* for 15 min. The resulting supernatant was then loaded onto a 5 mL HisTrap^TM^ FF column (Danaher Corporation, Washington, DC, USA) at 4 °C using an AKTA Pure FPLC system (GE Healthcare, Piscataway, NJ, USA). The His Tagged enzymes were eluted with His B buffer (50 mM Tris buffer, pH 7.5, 500 mM NaCl, and 250 mM imidazole) and further purified and buffer-exchanged with 20 mM Tris buffer, pH 7.5, 150 mM NaCl using a HiLoad 26/600 Superdex 200 pg size exclusion chromatography column (Danaher Corporation, Washington, DC, USA). The protein fractions were analyzed for purity using SDS-PAGE [36], concentrated with a 10 kDa cutoff spin concentrator (Sartorius, Stonehouse, UK), and flash-frozen as small aliquots in liquid nitrogen for long-term storage. The enzyme concentration was measured using absorbance at 280 nm with a NanoDrop^TM^ One Microvolume UV–Vis Spectrophotometer (Thermo Fisher Scientific, Waltham, MA, USA).

### 4.4. NOX Enzymatic Assay

The UV–Visible spectroscopic method for NADH quantification does not consider the biological activity of the NADH, so an enzymatic assay is necessary to validate the biological activity using the enzymatic consumption of NADH based on the loss of absorbance at 340 nm. The starting concentration of NOX was 29.43 mg/mL, and a dilution series was first performed with Tris pH 7.5 buffer to determine the necessary NOX concentration for use in an enzymatic assay with a 3 mL total cuvette volume and a concentration of 100 μM NADH. Using a 1:2000 dilution of NOX from the 29 mg/mL stock and a concentration of 50 to 100 μM NADH in a 1 cm path length cuvette containing Tris pH 7.5 buffer, the absorbance of the 340 nm peak was quenched in approximately 4 min. Dilution series were also performed in HEPES and phosphate buffers. Figure S2 shows the resulting performance curves at a NOX concentration of 150 ng/mL. Note that the NOX was first diluted to 15 μg/mL, and this solution was labeled “NOX dilutant”. For the enzymatic assay, the NADH solution was diluted to a concentration of approximately 100 μM in a 1 cm path length cuvette and analyzed either by scanning wavelength kinetics (i.e., 450 nm to 250 nm) (examples in Figure S2) or by continuous measurement of the absorbance at 340 nm. After one scan in the wavelength scanning kinetics studies, or after 30 s in the 340 nm continuous measurement study, the NOX dilutant was added to the cuvette, and the absorbance was measured for several minutes more. The typical cuvette contents were 2820 μL of 50 mM buffer, 150 μL of 2 mM NADH solution in buffer, and 30 μL of NOX dilutant (15 μg/mL).

## 5. Conclusions

Long-term stability testing of NAD^+^/NADH showed that 50 mM Tris buffer at pH 8.5 and 19 °C is a favorable aqueous buffer for storing NAD^+^/NADH solutions for 40+ days. Under these conditions, Tris buffer yields the lowest degradation rate for NADH (4 μM/d), with over 90% of the original NADH remaining when compared to the much faster degradation in HEPES (18 μM/d, 60% remaining active) and sodium phosphate (23 μM/d, <50% remaining active) buffers. Furthermore, the stability of NAD^+^ is qualitatively demonstrated, which is critical for systems in which turnover of the cofactors is desired. In the case of NAD^+^, Tris buffer was again found to be the most stable, but, in contrast to NADH stability, HEPES showed the poorest NAD^+^ stability. Lastly, NADH stability tests are supported by a NOX enzymatic activity assay, ensuring that the long-term stability of NADH in Tris buffer also maintains biological activity, and thus, the NADH concentration can be reliably monitored via UV–Vis under these conditions.

These findings are important in the efficient design of cofactor systems for cell-free bioreactors with and without cofactor regeneration. The NAD^+^/NADH cofactors are critical for many enzymatic reaction schemes, because many enzymes are highly specific to these cofactors. Currently, process efficiency and economics are major limiting factors for cell-free enzymatic reactions. Therefore, developing efficient and economical cell-free systems will require advances in the areas of cofactor recycling, stability, mimetics, and others. The findings reported here may serve as a basis for cofactor stability in common buffers as improvements in cofactor engineering are pursued. Further considerations for the application of the findings of this work to cell-free biocatalytic systems include the concentrations of cofactors warranted, solution conditions, including ionic strength and pH, and the activity of buffers as substrates for the enzymes of interest, among others.

## Data Availability

The original contributions presented in the study are included in the article/Supplementary Materials, and further inquiries can be directed to the corresponding author. The raw data will be made available by the authors on request.

## Supplementary Materials

The following supporting information can be downloaded at https://www.mdpi.com/article/10.3390/molecules29225453/s1: Figure S1: Example NADH calibration curve; Figure S2: NOX activity assay spectra.
