# Supplementary material for: Long-Term Stability of Nicotinamide Cofactors in Common Aqueous Buffers: Implications for Cell-Free Biocatalysis
Source: Molecules. 2024 Nov 19;29(22):5453. doi: 10.3390/molecules29225453 (PMC11597533; doi:10.3390/molecules29225453)
Supplement: Supplementary file 1 [file molecules-29-05453-s001.zip › molecules-3292247-supplementary.pdf]

Long-term Stability of Nicotinamide Cofactors in Common Aqueous Buffers: Implications for Cell Free Biocatalysis

Kody D. Wolfe [1], Markus Alahuhta [2], Michael E. Himmel [2], Yannick J. Bomble [2], G. Kane Jennings [3], and David E. Cliffel [4,*]

[1] Institute for Sustainable Energy & the Environment, Ohio University, Athens, OH
[2] National Renewable Energy Laboratory, Biosciences Center, Golden, CO
[3] Chemistry Department, Vanderbilt University, Nashville, TN
[4] Chemical & Biomolecular Engineering Department, Vanderbilt University, Nashville, TN
* Correspondence: d.cliffel@vanderbilt.edu

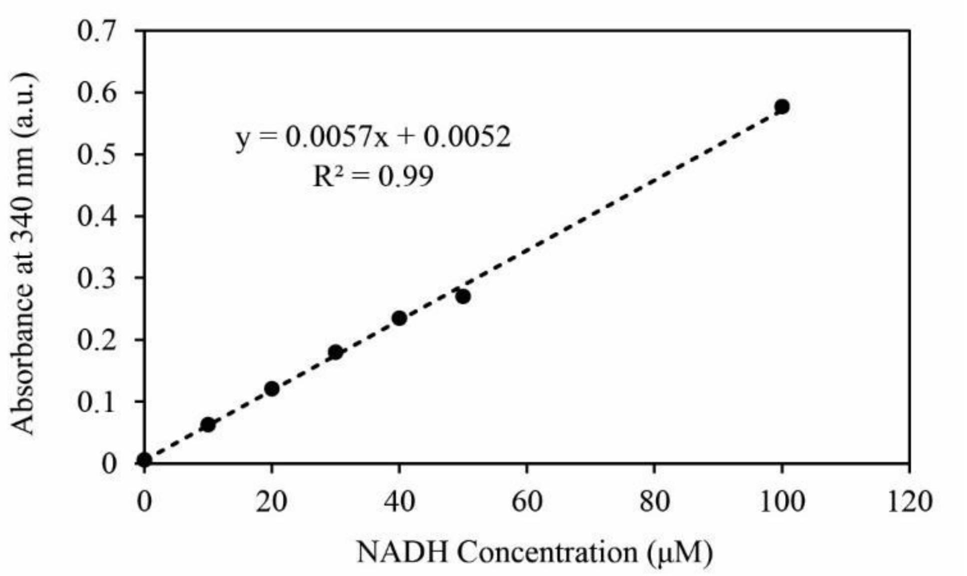

Figure S1. Calibration curve for absorbance at 340 nm vs. NADH concentration in HEPES buffer. Identical tests were completed in Tris and sodium phosphate buffers as well, resulting in minimally different slopes. These calibration curves were used to determine the NADH concentration used for stability curves shown in the following figures.

Amino Acid Sequence of *Lactobacillus brevis* NADH oxidase (UniProt: Q8KRG4) with C-terminal His-tag:

MGKVTVVGCTHAGTFAIKQILAEHPDAEVTVYERNDVISFLSCGIALYLGGKVADPQGL
FYSSPEELQKLGANVQMNHNVLAIDPDQKTVTVEDLTSHAQTTESYDKLVMTSGSWPI
VPKIPGIDSDRVKLCKNWAHAQALIEDAKEAKRITVIGAGYIGAELAEAYSTTGHDVTLI
DAMDRVMPKYFDADFTDVIEQDYRDHGVQLALSETVESFTDSATGLTIKTDKNSYETD
LAILCIGFRPNTDLLKGKVDMAPNGAIITDDYMRSSNPDIFAAGDSAAVHYNPTHQNAYI
PLATNAVRQGILVGKNLVKPTVKYMGTQSSSGLALYDRTIVSTGLTLAAAKQQGVNAE
QVIVEDNYRPEFMPSTEPVLMSLVFDPDTHRILGGALMSKYDVSQSANTLSVCIQNENTI
DDLAMVDMLFQPNFDRPFNYLNILAQAAQAKVAQSVNALEHHHHHH

https://www.uniprot.org/uniprotkb/Q8KRG4/entry

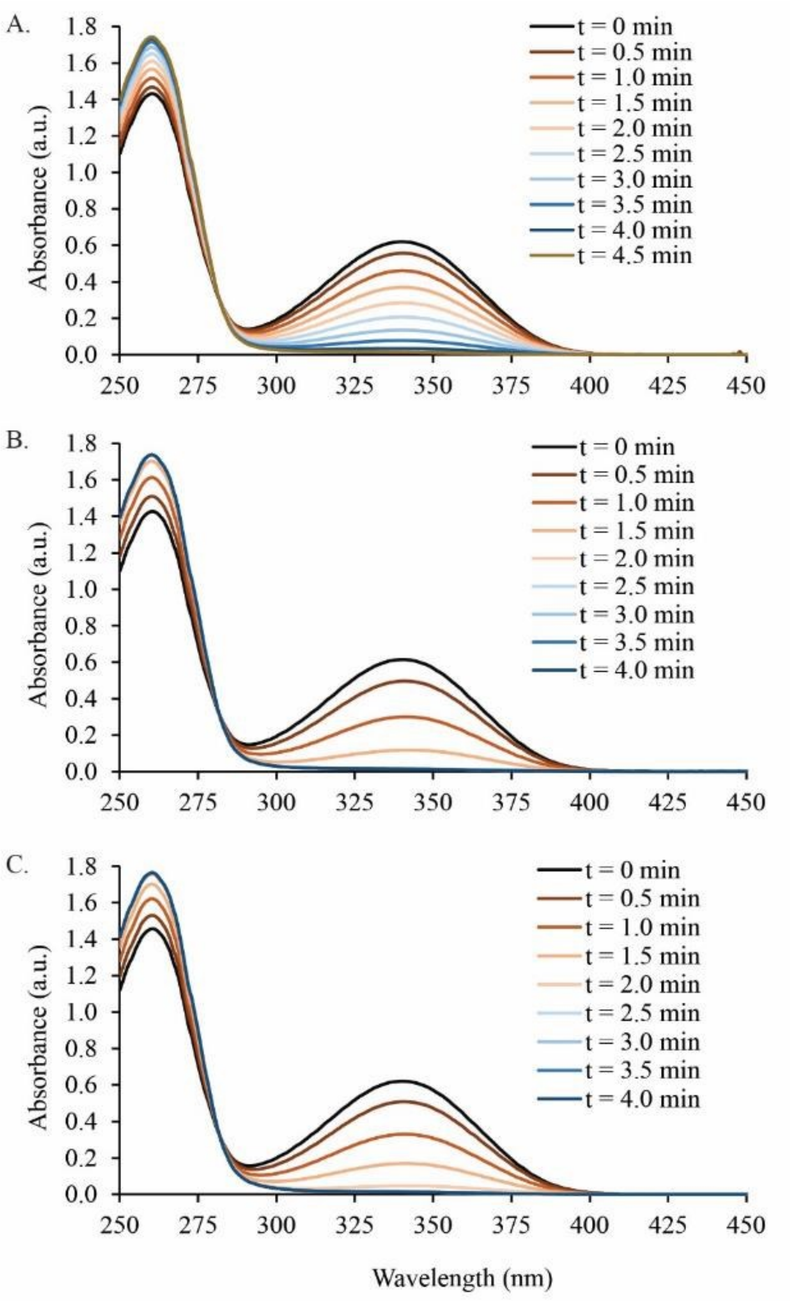

Figure S2. Examples (from dilution series study) of NOX consuming 100 uM NADH as shown by a decrease in absorbance at 340 nm wavelength in A) Tris, B) HEPES, and C) phosphate buffers at pH 8.5 and buffer concentration of 50 mM.
